# Long-Term SARS-CoV-2 Findings Related to Persisting Viral Antigen and Inflammation Resemble Those Reported for Influenza Virus and Respiratory Syncytial Virus

**DOI:** 10.3390/v16091353

**Published:** 2024-08-24

**Authors:** Norbert J. Roberts

**Affiliations:** 1 Division of Infectious Diseases and Immunology, Department of Medicine, New York University Grossman School of Medicine, New York, NY 10016, USA; norbert.roberts@nyulangone.org; 2 Division of Infectious Diseases, Department of Internal Medicine, University of Texas Medical Branch at Galveston, Galveston, TX 77555, USA

**Keywords:** SARS-CoV-2, influenza virus, respiratory syncytial virus, viral antigen, viral RNA, persisting inflammation, asthenia

## Abstract

Recent studies have documented prolonged expression of viral antigens and RNA and associated inflammation after infection with SARS-CoV-2 in a substantial proportion of infected patients. The persisting SARS-CoV-2 effects and findings, with inflammation associated with continued detection of viral antigens, especially resemble those previously reported for influenza virus, as well as respiratory syncytial virus (RSV). The reports indicate the need for improved insight into the mechanisms whereby post-SARS-CoV-2 infection-related illness is apparently more common and perhaps even more persistent after infection than observed for other respiratory viruses.

## 1. Introduction

Post-acute infection syndromes (PAISs) are currently documented in a minority of patients infected by respiratory viruses or other infectious agents [1], and it is likely that many such episodes are unrecognized [2]. Long COVID, a PAIS occurring after acute SARS-CoV-2 infection, has raised awareness of PAISs but is clearly not unique in producing such sequelae. It may, however, be more common and more pronounced than PAISs due to other respiratory viruses [3].

In an important July 2024 research article, Peluso and colleagues reported the results of their examinations of a cohort of 24 individuals at time points ranging from 27 to 910 days after acute SARS-CoV-2 infection [4]. The studies followed their earlier findings of prolonged changes in T lymphocytes after the acute infection [5], as well as reports of persisting viral antigens for months after acute infection by other investigators, in references cited by Peluso and colleagues, as well as reviewed by others [6]. They traced activated T lymphocytes, finding them in many regions, with tracer uptake in lung tissue higher in those with persistent pulmonary symptoms specifically. However, increased T cell activation in the tissues was also observed in many individuals without long-term sequelae. In a subset of subjects, they found intracellular viral RNA in biopsied gastrointestinal tract tissue, measured because of high tracer activity. They rightly concluded that tissue viral persistence could be associated with long-term immunological perturbations [5,7].

The persisting SARS-CoV-2 effects and findings, with inflammation associated with continued detection of viral antigens, resemble those previously reported for influenza virus, especially, as well as respiratory syncytial virus (RSV). It is notable that PAISs are associated with SARS-CoV-2, influenza virus, and RSV, which are detectable in previously healthy individuals for far shorter periods after infection than rhinovirus or bocavirus [8,9]. Rhinovirus can persist in both immunocompetent and immunosuppressed individuals and can cause lower respiratory tract infection, episodes of asthma, and exacerbation of chronic obstructive pulmonary disease [8,10], but it has not clearly been associated with PAISs.

## 2. Evidence of Persisting Influenza Virus Antigens and Associated Inflammation after Acute Infection

Asthenia has long been recognized as a major process associated with influenza virus infection. In 1919, Cowie and Beaven remarked on its presence and severity during the recent pandemic [11], noting the following: “No matter how mild the attack, asthenia was almost invariably one of the most, if not the most, prominent symptom. It was usually out of all proportion to the other symptoms and persisted not only during the attack but far into convalescence”.

One of the less recognized features of influenza virus infection is that pathological and immunological changes in the lung can persist long after apparent clearance of the virus [12]. The possible connection of such long-term effects after the acute infection to the persistence of viral antigens and inflammation was raised >40 years ago by Jakab and colleagues in studies using a murine model of influenza virus infection [13]. Lavageable lung cell populations and differential counts were quantitated from 3 days to a year after the acute infection. An expected inflammatory cellular response during the acute phase of the infection did not completely resolve, with the pulmonary leukocytosis remaining stable from day 30 through a year after the acute virus infection. Histopathologic examination of virus-infected lungs showed an ongoing inflammatory response. The infectious virus could not be recovered after day 9, but the viral antigen persisted at high concentrations in the lungs. The investigators concluded that influenza virus infection induced long-term alveolitis, which is associated with the persistence of viral antigens [13].

Other studies in mice demonstrated the long-lasting existence of influenza-induced inflammation [14]. Three weeks following infection, mice showed increased numbers of CD4^+^ and CD8^+^ T cells and histopathological changes. Transcriptomics revealed increased expression of proinflammatory and profibrotic genes that persisted for several weeks following the infection [14]. Viral clearance was confirmed by RT-PCR on day 21 after influenza infection, but the mice maintained elevated inflammatory cell counts in the bronchoalveolar lavage (BAL) samples. There was persistent lung inflammation, epithelial injury, metaplasia, and genetic reprogramming in the weeks after the primary influenza infection. Specifically, ER stress and transcriptomic and epigenetic changes remained substantially after viral clearance and recovery from disease-induced morbidity.

Zammit and colleagues [15] reported that activated virus-specific CD8 T cells remain in the lung airways for several months after influenza virus infection. They showed that maintenance of that cell population was dependent upon the route of infection and prolonged presentation of viral antigen in the draining lymph nodes (DLNs) of the respiratory tract. Their data showed that the antigen that is retained after pulmonary influenza virus infection controls the migratory pattern and activation state of virus-specific CD8 T cells near the site of virus amplification for at least 2 months after infection. The CD8 effector T cell population in the lung airways continued to be replenished by migrating T cells in the circulation.

Keeler and colleagues [16] showed that influenza A virus infection upon intranasal delivery of the virus achieved a marked replication and increase in viral load in the lung as well as a dose-dependent severity of acute illness in mice. These findings were followed by evidence of the persistence of negative- and positive-strand viral RNA remnants for 15 weeks and chronic lung disease for at least 26 weeks post-infection. The disease was manifested by focal areas of bronchiolization and mucus production that contained increased levels of viral RNA remnants. Despite clearance of infectious virus by 12 days post-infection, based on culture and viral immunostaining, virus-specific RNA was detectable in the lung even at 105 days post-infection. Influenza virus RNA also persisted in lung lymph nodes at 77 days and spleen at 49 days post-infection. The results of such murine model studies suggest that a human respiratory virus could produce chronic lung disease focally at sites of active viral RNA remnants.

Huckestein and colleagues [17] reported that persistent inflammation following severe influenza infection in mice was primarily driven by macrophages and T cells. They cited observations that proinflammatory and profibrotic gene expression remains elevated in mice for up to 60 days after infection and that associated lung remodeling is characterized by airway occlusion, epithelial honeycombing, and alveolar cysts [14]. All the studies cited by these authors and described in this section, as well as clinical observations, indicate that seasonal and pandemic influenza virus infection can cause persistent lung injury following viral clearance. The authors specifically commented that the recent development of long COVID-19 in patients with COVID-19 who experienced a range of acute disease severity illustrates the risk of prolonged viral antigen persistence and associated inflammation during pandemic outbreaks driven by respiratory tract viruses.

Wong and colleagues compared the immune responses of influenza-confirmed hospitalized individuals to severe acute respiratory illness (SARI) to those of nonhospitalized individuals with influenza-like illness (ILI) [18]. Peripheral blood lymphocytes were collected from 27 patients with ILI and 27 with SARI at the time of enrollment and then 2 weeks later. By the convalescent phase, most SARI cases displayed continued immune activation characterized by increased numbers of CD16+ monocytes and proliferating influenza virus-specific, CD8+ T cells as compared with ILI cases. SARI was also associated with reduced amounts of cytokines that regulate T-cell responses (i.e., interleukin 4, interleukin 13, interleukin 12, interleukin 10, and tumor necrosis factor β) and hematopoiesis (interleukin 3 and granulocyte-macrophage colony-stimulating factor) but increased amounts of a proinflammatory cytokine (tumor necrosis factor α), chemotactic cytokines (MDC, MCP-1, GRO, and fractalkine), and growth-promoting cytokines (PDGFBB/AA, VEGF, and EGF) compared with ILI. The authors concluded that severe influenza cases showed a delay in peripheral immune activation that likely led to prolonged inflammation compared with mild influenza cases.

## 3. Evidence of Persisting Respiratory Syncytial Virus Antigens and Associated Inflammation after Acute Infection

Bermejo-Martin and colleagues studied the patterns of expression of 27 immune mediators during the recovery process from severe RSV infection and after complete resolution of symptoms [19]. They recruited 37 hospitalized children younger than 2 years of age and reported several patterns of immune mediator responses. Although one pattern of the children’s responses indicated a decrease in mediators from admission to discharge, the other three patterns of immune mediator responses revealed that certain proinflammatory responses induced by RSV persist well beyond the resolution of symptoms because all but one of the mediators included in those patterns mediate or facilitate migration of inflammatory cells to the focus of infection. Unfortunately, the number of subjects exhibiting each pattern was not indicated. Nonetheless, they documented the persistence of proinflammatory responses in the long term in fully recovered patients with RSV [19].

Estripeaut and colleagues characterized the significance of RSV RNA persistence and its relation to RSV-induced chronic airway disease [20]. Mice were inoculated with medium or with live RSV or inactivated virus preparations. Bronchoalveolar lavage fluid samples were obtained, and lung specimens were harvested on days 1, 5, and 42 after inoculation to assess lung inflammation and lung mRNA expression of an array of immunologics; RSV loads were assessed using culture and real-time polymerase chain reaction (PCR) and were correlated with pulmonary function. During the acute phase of infection, RSV loads, as indicated by culture and PCR, were significantly higher in mice inoculated with live RSV, as would be expected. On day 42, RSV RNA remained detectable in mice inoculated with live RSV. Lung inflammation, airway obstruction (AO), and airway hyperreactivity (AHR) were significantly increased in mice inoculated with live RSV. AO on day 5 and AHR on day 42 were significantly correlated with RSV RNA copy number in lung samples.

Schwarze and colleagues challenged mice intranasally with human RSV, analyzed sequential tissue samples by direct culture and polymerase chain reaction for viral and messenger RNA, and monitored antiviral immune responses [21]. The virus could not be detected in bronchoalveolar lavage samples beyond day 14, but viral genomic and messenger RNAs were present in lung homogenates for 100 days or more. The combined depletion of CD4 and CD8 T cells allowed the infective virus to be recovered. Thus, they presented evidence of RSV latency and persistence in the lungs despite RSV-specific antibody and cytotoxic T lymphocyte (CTL) activity against a major RSV epitope.

## 4. Concluding Remarks

Relatively early in the COVID-19 pandemic, Salamanna and colleagues reviewed the development of long COVID in SARS-CoV-2-infected patients [22], with symptoms lasting for months. Their extensive review indicated that long-term issues included abnormal lung functions, neurological complaints, olfactory dysfunctions, and widespread symptoms such as chronic fatigue and pain. Their report thus indicated that a proportion of patients who had been infected by the SARS-CoV-2 virus developed a post-COVID syndrome. In fact, as recently reported by Fang and colleagues [23], approximately 7% of infected U.S. patients have experienced long COVID.

Kanth and colleagues [24] assessed homeostatic mechanisms in the lung after COVID-19, measuring changes over a year in the protein signature of bronchoalveolar lavage from 45 patients with mild to moderate disease who recovered at three phases (acute, recovery, and convalescent). In contrast to healthy persons, the post-COVID-19 lung proteome revealed persistent elevations of proteins associated with tissue repair and activation of proteostasis up to 9 months after symptom onset. Notably, these processes were still occurring despite the resolution of clinical and radiographic signs of the prior infection. Serial follow-up suggested that the pulmonary proteome did not return to normal for almost one year after the onset of COVID-19. Therefore, there was ongoing host repair in response to the initial infection, even in patients with mild to moderate infections who had clinically recovered. The authors noted that a detailed proteomic analysis of other viral pulmonary infections after recovery is needed.

Treatment of PAISs such as long COVID is actively being investigated, for example, with licensed antiviral agents such as nirmatrelvir-ritonavir, without finding significant benefit for symptoms [25]. Alternate approaches are being developed, such as the use of symbiotics [26], with the first results being promising. In addition, attempts are being made to identify effective non-pharmacological therapies for PAISs, including long COVID [27,28], with multidisciplinary teams vital for the effort [27,29]. It is important to emphasize that perhaps the most effective approach to PAISs is prevention. Licensed vaccines are available for SARS-CoV-2, influenza virus, and RSV. Vaccination should be strongly encouraged to address the major burden of PAISs on patients, their families, and the health care system.

As reviewed briefly above, the findings of prolonged viral antigen or RNA expression and associated inflammation and repair after infection with SARS-CoV-2 resemble those recognized for subsets of patients or animals in studies of infection by influenza virus or respiratory syncytial virus. Thus, what is needed is improved insight into the mechanisms whereby post-SARS-CoV-2 infection-related illness is apparently more common and perhaps even more persistent after infection than observed for other respiratory viruses. The results of such investigations may enhance medical intervention to limit such findings for the next respiratory virus pandemic.

## Data Availability

Not applicable.
